# The Role of Biomarkers in Monitoring Chronic Fatigue Among Male Professional Team Athletes: A Systematic Review

**DOI:** 10.3390/s24216862

**Published:** 2024-10-25

**Authors:** Alejandro Soler-López, Adrián Moreno-Villanueva, Carlos D. Gómez-Carmona, José Pino-Ortega

**Affiliations:** 1 Department of Physical Activity and Sport, Faculty of Sport Sciences, University of Murcia, 30720 San Javier, Spain; josepinoortega@um.es; 2 BioVetMed & SportSci Research Group, University of Murcia, 30100 Murcia, Spain; adrian.moreno@ui1.es; 3 Faculty of Health Sciences, Isabel I University, 09003 Burgos, Spain; 4 Research Group in Optimization of Training and Sports Performance (GOERD), Department of Didactics of Music Plastic and Body Expression, Faculty of Sport Science, University of Extremadura, 10003 Caceres, Spain; 5 Department of Music, Plastic and Body Expression, Faculty of Human and Social Sciences, University of Zaragoza, 44003 Teruel, Spain

**Keywords:** physiological load, training adaptation, muscle damage, immune markers, hormonal responses

## Abstract

This systematic review synthesizes evidence on biomarker responses to physiological loads in professional male team sport athletes, providing insights into induced fatigue states. Structured searches across major databases yielded 28 studies examining various biomarkers in elite team sport players. Studies evaluated muscle damage markers, anabolic/catabolic hormones reflecting metabolic strain, inflammatory markers indicating immune activity and tissue damage, immunological markers tied to infection risk, and oxidative stress markers showing redox imbalances from excessive physiological load. Responses were examined in official matches and training across competitive seasons. The evidence shows that professional team sports induce significant alterations in all studied biomarkers, reflecting measurable physiological strain, muscle damage, oxidative stress, inflammation, and immunosuppression during intensive exercise. These effects tend to be larger and more prolonged after official matches compared to training. Reported recovery time courses range from 24-h to several days post-exercise. Monitoring biomarkers enables quantifying cumulative fatigue and physiological adaptations to training/competition loads, helping to optimize performance while mitigating injury and overtraining. Key biomarkers include creatine kinase, testosterone, cortisol, testosterone/cortisol ratio, salivary immunoglobulin-A, and markers of inflammation and oxidative stress. Further research should extend biomarker monitoring to cover psychological stress and affective states alongside physiological metrics for deeper insight into athlete wellness and readiness.

## 1. Introduction

Achieving optimal performance while minimizing injury risk in team sport athletes requires balancing between training load (TL) and recovery [1,2,3]. The training program aims to enhance performance by gradually increasing load, disrupting athletes’ internal equilibrium [4,5]. However, professional sports demand that athletes achieve peak performance within a limited timeframe or over an extended period of time [5]. As a result, high TL is utilized during the preparation period to achieve performance gains [6]. Coaches employ planning, monitoring, and organizational strategies to manage the training program and evaluate athletes’ responses [7]. Despite available knowledge, there remains a limited understanding of the specific interactions between TL, resultant fatigue response, and subsequent performance. In this context, beyond the standard parameters of performance and load management such as HRV (heart rate variability) [8,9], RPE (rate of perceived exertion) [6,10], Linear Position Transducers and Linear Velocity [11,12,13], or tracking systems [14,15], it is imperative to consider the integration of biochemical markers. These assessments are crucial in assessments to help prevent imbalances and overtraining during congested schedules [16]. Therefore, successful training planning in team sports relies on the accurate monitoring and interpreting of training adaptations using objective data on physical performance, biochemical markers, and physiological variables [1,17,18,19].

The sports science literature has extensively investigated the impact of TL on various biochemical markers that reflect physiological stress and recovery [20,21,22]. Several biochemical markers such as creatine kinase (CK), C-reactive protein (CRP), and creatinine have been linked to exercise-induced muscle damage and used to quantify biochemical responses to TL changes [23,24]. However, evidence for CK changes with acute or chronic TL remains moderate [24], and its use in TL monitoring is still under debate due to variability in CK activity based on exercise type, intensity, duration, and evaluation time [20,23]. On the other hand, testosterone, cortisol, and the testosterone/cortisol ratio (T/Cr) are other biochemical markers associated with TL-induced stress [20,25,26]. These hormonal markers reflect the metabolic adaptations and recovery responses to TL across seasons for specific sports [27]. While alterations in testosterone and cortisol levels caused by chronic training remain unclear, the T/Cr ratio changes show moderate evidence [24]. Salivary immunoglobulin-A (s-IgA) and *α*-amylase (s-AA) are other biomarkers of interest, which are antimicrobial proteins secreted by mucosal cells under sympathetic nervous system (SNS) control [28]. These markers have been used to track TL changes in soccer players and athletes, as their stress-related secretion indicates acute stress [28,29,30]. However, in response to prolonged stressful stimuli or increased physical training demands, a reduction in s-IgA and s-AA occurs, which is associated with an increased risk of upper respiratory tract infection (URTI) and symptoms (URTSs) in soccer players [31,32].

The scientific literature is increasingly recognizing sport as a complex psycho-physiological activity, wherein even minor TL fluctuations significantly influence athletes’ physical performance, stress levels, and wellness status [2,33]. Consequently, several studies have emphasized biomarkers’ utility for monitoring training-related stress, strain, recovery, and wellness to identify early signs of fatigue and potential overtraining in high-performance sports programs. However, it is important to note that, as of now, there is no systematic review addressing the most used biomarkers to detect fatigue in professional team athletes. Reviews to date primarily focus on narrow areas: specifically, soccer [34,35,36], indoor sports [37], and team ball sports that include both professional and amateur levels [38]. This absence highlights a critical gap in the literature, underscoring the need for this systematic review to synthesize and evaluate the existing evidence on biomarkers in professional sports settings. Therefore, the aim of this study is to determine the primary biomarkers used in professional team sport athletes for detecting fatigue arising from training or match loads.

## 2. Materials and Methods

### 2.1. Design

The present study was a systematic review conducted following the PRISMA (Preferred Reporting Items for Systematic Reviews and Meta-Analyses) protocol [39,40]. PRISMA allows for synthesizing the most relevant information on a topic to make it more practical and applicable, providing readers with up-to-date and useful information on a constantly evolving research area.

### 2.2. Search Strategy

**Figure 1:**
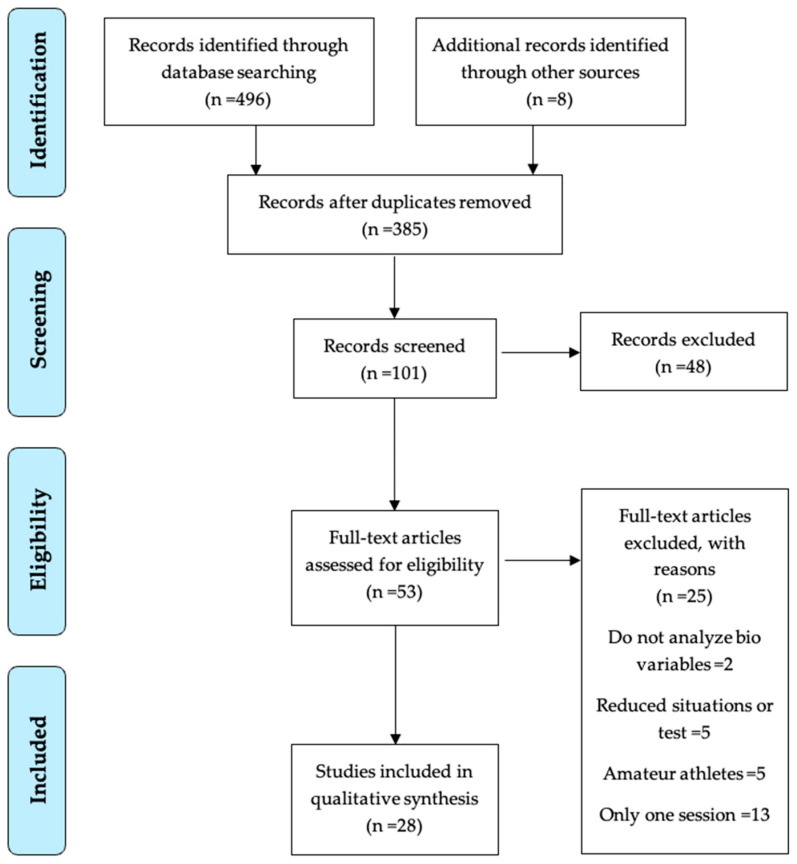
PRISMA flow diagram.

For this systematic review, we consulted the following electronic databases: PubMed, Scopus, SportDiscus, and Web of Science. We selected these databases as they are comprehensive resources that index the sports science literature, enabling access to domain-specific articles relevant to the review topic [41]. The search was conducted on 22 December 2023 using Boolean operators “AND” and “OR” to combine the keywords: “ (“elite” OR “professional”) AND (“team sport*”) AND (“physiological” OR “immunological” OR “biochemical” OR “hormonal”) AND (“fatigue” OR “performance” OR “recovery” OR “stress” OR “wellness”)”. Figure 1 presents the search process results via a flowchart. We also reviewed the reference lists of the included studies to identify additional relevant articles. Any disagreements regarding study inclusion were resolved by consensus between two investigators (A.S.-L. and C.D.G.-C.) and arbitration by a third investigator (J.P.-O.) when needed.

### 2.3. Inclusion and Exclusion Criteria

The selection of studies for this review was based on specific criteria related to biomarker reporting and measurement. The inclusion criteria for articles were (1) studies reporting on at least one of the following categories of biomarkers: (a) muscle anabolic/catabolic hormones (e.g., testosterone, cortisol), (b) muscle damage markers (e.g., creatine kinase, lactate dehydrogenase), (c) immunological markers (e.g., salivary immunoglobulin A, immune cell function), (d) oxidative stress markers (e.g., reactive oxygen species, antioxidant capacity), and (e) inflammatory markers (e.g., C-reactive protein, cytokines); (2) a clear description of biomarker acquisition methods, including (a) sample type (e.g., blood, saliva, urine) (b) sampling time points (e.g., pre-exercise, post-exercise, during recovery), and (c) analytical techniques used (e.g., ELISA, spectrophotometry); (3) studies conducted on elite or professional male team sport athletes; (4) biomarker data collected from official matches and/or training sessions; and (5) longitudinal studies or those analyzing more than one official competition match or training session.

On the other hand, the exclusion criteria were (1) studies on amateur or youth athletes; (2) laboratory-based or simulated exercise scenarios; (3) studies that did not provide adequate details on biomarker measurement methods; (4) single time-point measurements without consideration of changes over time; (5) studies focusing solely on biomarkers not directly related to fatigue or recovery (e.g., nutritional markers); and (6) documents such as theses, books, or systematic reviews (excluded only as a bibliographic source, not from systematization). The minimum publication year was 2000, as earlier reviews noted this as the starting point.

### 2.4. Screening Strategy and Study Selection

One investigator (A.S.-L.) conducted searches, identified relevant studies, and extracted data in a standardized, disaggregated manner. The review process followed Prisma guidelines [39] and recommendations for sports science systematic reviews [41] (Figure 1). The extracted articles were organized via a Microsoft Excel (version 16.78, Microsoft, Redmond, WA, USA) database detailing the database, keywords, article identifiers, and publication year. The articles were reviewed, and duplicates were eliminated. Then, the titles and abstracts of the remaining articles were read, and those unrelated to the topic were discarded. When necessary, the full text was read to verify compliance with the eligibility criteria and judge the relevance of the article. After this process, a total of 28 articles were selected. The data were analyzed and tabulated considering contextual variables such as the type of sport (soccer, basketball, volleyball, or handball), the type of event (matches or training), and the type of bio-measured variable (physiological, immunological, biochemical, or hormonal).

### 2.5. Quality of Studies

Two authors (A.S.-L. and C.D.G.-C.) assessed the risk of reporting bias via the Methodological Index for Non-Randomized Studies (MINORS) checklist [42]. MINORS has twelve items, four of which are only applicable to comparative studies. Each item is scored 0 when the criterion is not reported in the article, 1 if it is reported but not sufficiently met, or 2 when it is adequately met. Higher scores indicate a good methodological quality of the article and a low risk of bias. Therefore, the highest possible score is 16 for non-comparative studies and 24 for comparative studies. MINORS has provided acceptable inter and intra-rater reliability, internal consistency, content validity, and discriminant validity [42,43].

## 3. Results

### 3.1. Identification and Selection of Studies

After conducting the search, 504 relevant studies were initially found (496 databases and 8 additional records through other sources). Once duplicate studies were eliminated, there remained 385 unique studies to review. The titles and abstracts of these 385 studies were screened, leading to the identification of 53 potentially eligible studies. The remaining studies were excluded due to their lack of relevance to the subject matter of the manuscript. The full texts of these 53 studies were retrieved and inspected against the inclusion/exclusion criteria. This full-text review process filtered out 25 studies that did not satisfy the criteria. Ultimately, 28 studies successfully were selected. The process of searching, identifying, and selecting the studies is illustrated in Figure 1.

### 3.2. Methodological Quality

The results of the methodological risk of bias of the articles included in this review can be found in Table 1. From the total 28 studies, 13 studies are comparative (24 maximum points) and 15 are non-comparative (16 maximum points). Nineteen studies present a low risk of bias with B Score (two comparative and eleven non-comparative studies). No study has an A score. Four comparative studies present a high risk of bias (C Score). The worst evaluated item in all types of studies is item 5 (Evaluations carried out in a neutral way), while the worst evaluated item in comparative studies is item 8 (A control group having the gold standard intervention).

### 3.3. Characteristics of the Selected Studies

Table 2 shows the characteristics of the selected studies in the present systematic review. The included studies ranged from 2008 to 2023. The earliest study was published in 2008, while over 70% of the studies (*n* = 16) emerged from 2015 onwards, highlighting the growing research attention on this topic.

The selected studies involved elite athletes from different team sports. The most evaluated sport was basketball (*n* = 7) [53,56,62,63,64,65,67], followed by soccer (*n* = 6) [57,58,60,61,64,66], handball (*n* = 3) [47,55,64], futsal (*n* = 3) [20,44,59], rugby (*n* = 3) [30,54,69], Australian football (*n* = 3) [48,49,50], volleyball (*n* = 2) [52,64], rugby union (*n* = 2) [51,68], netball (*n* = 1) [45], and water-polo (*n* = 1) [46].

Regarding the context of evaluation, eight studies analyzed responses to official matches [45,48,49,54,58,60,64,69], eight studies focused exclusively on regular training [20,46,47,51,52,59,67,68], and twelve studies examined both matches and training workloads [30,44,50,55,56,57,61,62,63,65,66,68]. When comparing official matches with training sessions, official matches impose greater physiological demands, which provoke heightened stress responses.

The most frequently assessed biomarkers were muscle anabolic/catabolic hormones (testosterone and cortisol) (*n* = 15) [30,45,46,50,52,53,55,57,58,60,63,64,66,70,71], damage markers (creatine kinase and lactate dehydrogenase) (*n* = 9) [20,44,45,52,55,57,61,64,69], immunological markers (Immunoglobulin A and immune cell function) (*n* = 8) [46,48,49,51,54,59,66,68], oxidative stress markers (reactive oxygen species and antioxidant capacity) (*n* = 6) [44,47,55,57,65,67], and inflammatory markers (C-reactive protein and cytokines) (*n* = 4) [47,57,61,64].

## 4. Discussion

This study aimed to systematize and analyze the existing scientific evidence on the primary biomarkers most frequently used in professional team athletes to detect the fatigue induced by the physical demands of professional training and competition. This physical and physiological stress is a direct response to exercise that can be experienced during both training and competition and leads to elevated levels of fatigue [38]. The multifactorial and complex nature of fatigue necessitates a comprehensive analysis of various biomarkers, as summarized in Table 3. This table provides an overview of the key biomarker categories, including muscle anabolic/catabolic hormones, muscle damage markers, immunological markers, oxidative stress markers, and inflammatory markers, along with their relevance to chronic fatigue assessment and typical measurement methods. By examining these diverse biomarkers, we can gain a more holistic understanding of the physiological responses to training and competition loads. This rigorous examination is vital to ascertain the extent of athletes’ physiological adaptation, effectively curtailing the risks associated with non-functional overtraining, injury, or disease linked to prolonged fatigue accumulation [2]. The analysis of these biomarkers, in conjunction with workload data, provides a more comprehensive approach to monitoring and managing athlete fatigue in professional team sports [2].

### 4.1. Hormonal Markers

The relationship between hormonal markers and training/competition loads was evaluated in 15 of the included studies [30,45,46,50,52,53,55,57,58,60,63,64,66,70,71]. Regarding the impact of training load (TL) and competition loads (CL) on hormonal responses, all studies included in this review show significant alterations in testosterone, cortisol, and the testosterone/cortisol ratio in response to changes in external and internal TL/CL across the season. These hormonal perturbations provide useful information for athlete-monitoring purposes to detect dysfunctional physiological responses. Emphasizing the practical significance of these findings, the T/C ratio has emerged as a particularly sensitive marker in gauging training stress and fatigue levels. A study performed on rugby players indicated that only cortisol levels present limitations as a physiological stress biomarker due to their variability, indicating that the combination with testosterone values provides a more reliable index [30]. In this way, Schelling et al. [63] obtained that the hormonal status varies according to playing position and game time, impacting training and recovery strategies. The diversity in findings, as collated in a recent systematic review by Moreno-Villanueva et al. [37], corroborates earlier hypotheses about the variability of T, C, and T/C marker values [20,30,62,63]. This variability is contingent on the period under analysis and the specific sporting discipline. While some researchers advocate for the use of T and C as individual indicators of fatigue in team sports [20], the complexity inherent in the hormonal response necessitates a broader investigation into the interplay between these hormones, particularly through the lens of the T/C ratio. This approach allows for a nuanced and comprehensive interpretation of the data, shedding light on the balance between anabolic and catabolic processes. However, it is crucial to acknowledge that these hormonal parameters should not be isolated in their interpretation. This observation facilitates the precise calibration of training regimens through the utilization of hormonal biomarker data, with the objective of achieving optimal performance enhancement. Therefore, appropriately adjusting training and recovery programs based on hormonal biomarker data can aid performance optimization.

### 4.2. Muscular Damage Markers

Substantial research evidences the pattern of consistent CK elevation post-exercise that induces fatigue and muscle damage [72,73,74,75]. Our review corroborates this, indicating notable sustained elevations in CK levels [20,44,45,52,55,57,61,64,69]. However, several aspects merit consideration in the interpretation of our data. Primarily, prior studies have revealed considerable day-to-day fluctuations in CK levels [45,57,64,69]. These findings are further supported by other studies [76,77], which also observed an approximate change of around 26–27%, respectively, with the smallest worthwhile change (SWC) in CK identified at 8.6% [76]. Ideally, the smallest worthwhile change (SWC) should be less than the coefficient of variation for effective sensitivity. However, based on the results of the indexed studies [45,57,64,69], the post-match increases were significantly greater (*p* < 0.05) than the athletes’ coefficients of variation, with a significant increase (*p* < 0.05) in CK levels compared to the average baseline level at 24, 48, and 72 h. Therefore, it appears that the use of CK as an indicator of muscle damage is a sensitive tool for detecting the acute load borne by athletes [45,57,64,69,76,77].

Although it is undeniable that CK levels rise following intense exercise, its effectiveness as a measure to monitor an athlete’s chronic load seems to be less reliable [20]. Nonetheless, the study reported by Barcelos et al. [44] and Marin et al. [55] demonstrates how CK and Lactate Dehydrogenase (LDH) can be a sensitive tool for detecting changes in muscle damage levels over the preseason and season. Discrepancies among study results can be explained by several factors [20,44,55]: it is important to note how much time elapsed since the last training or match for CK measurements because if measurements are taken within the first 78 h, CK levels after an intense match or training will be significantly higher than baseline. However, after these 78 h, CK levels are less effective in detecting muscle damage as shown in the study by Birdsey et al. [45], with the highest blood CK levels recorded at 24–48 h after a training session or match in professional athletes [45,57,64,69]. Another consideration is the circadian fluctuation of CK; under typical resting conditions, CK concentrations peak in the morning [78], which can influence the timing of measurement. In our review, sampling times varied greatly; however, it is likely that the substantial increases in CK after training or matches (24–72 h) overshadow this variation [45,57,64,69]. Finally, it is important to highlight at what point in the season CK and LDH samples were taken. While the study reported by Miloski et al. [20] showed no significant differences in blood CK during the season, it did reveal significant changes (*p* < 0.05) in CK (266 μ/L) during the preseason when the training load was higher. Similar results were shown in the studies by Barcelos et al. [44] and Marin et al. [55], where significant differences in CK and LDH were found when there was a significant reduction in training load as a strategy to improve team performance [44] and during periods of match congestion and intensity such as the playoff season [45,52,55,57,61].

In this context, CK appears to be a sensitive marker for detecting muscle damage in professional athletes. It is essential to consider that CK levels undergo circadian fluctuations, generally being higher in the morning. This should be taken into account when assessing CK levels for accurate results. Moreover, this enzyme shows greater sensitivity 72 h post-training or competition, reaching its peak between 24 and 48 h after intense physical activity [45,57,64,69]. Therefore, measuring CK levels during these periods can provide valuable information about an athlete’s muscular state. Additionally, CK and LDH can be a useful indicator for monitoring variations in physical state during the season, especially during periods of congested matches or a decrease in training and/or match load. This tool enables coaches and athletes to adjust their training and recovery programs more effectively, minimizing injury risk and optimizing performance.

### 4.3. Immunological Markers

s-IgA has emerged as a pivotal biomarker for evaluating overtraining, psychological stress, and the health status of the upper respiratory tract, as underscored in seminal research [79]. s-IgA predominantly functions as a barrier against viral infections, obstructing the adherence of pathogens to the mucosal epithelium of the upper respiratory tract, a mechanism well-documented in the work of Rico-González et al. [79]. Notably, an escalation in training intensity can precipitate a decline in s-IgA levels, augmenting the vulnerability to upper respiratory tract infections (URTI), as elucidated in various studies [80]. This systematic review scrutinizes eight studies that investigated s-IgA responses to structured training and competitive engagements. These studies delve into the dynamics of s-IgA and other immunological markers (salivary lysozyme, neopterin, and total neopterin) under varying training modalities, encompassing periodization, overload, tapering, and preparatory phases [46,48,49,51,54,59,66,68].

A subset of the reviewed literature established correlations between s-IgA concentrations and URTI prevalence [51,59,68]. Moreira et al. [59] conducted a nuanced analysis over a four-week intensive training period, hypothesizing and confirming a negative correlation between escalated training loads and s-IgA levels, with a concomitant increase in URTI symptoms, particularly pronounced in the final week. This finding highlights the susceptibility of athletes with reduced s-IgA to URTI risks. Complementarily, another study identified a low s-IgA secretion rate as a risk factor for URTI [81], while also noting the contribution of heightened training load and intensity to URTI incidence [81,82]. Further corroborating this, the study by Tiernan et al. [68] demonstrated that a reduction of ≥65% in s-IgA levels significantly escalated the risk of URTI in the ensuing two weeks. On the other hand, another study [51] reported no substantial correlations between absolute s-IgA or salivary lysozyme (s-Lys) levels and URI incidence. However, they observed a trend of lower s-IgA concentrations in players with higher URTI instances compared to asymptomatic players, indicating a potential association between diminished s-IgA levels and elevated URTI risk. Notably, the study also revealed position-specific variations in s-IgA and s-Lys levels, and URI incidence, underscoring the importance of maintaining optimal s-IgA levels to mitigate URTI risks.

Another salient outcome from this systematic review is the association between increased training loads and decreased s-IgA levels. As Botonis and Toubekis [46] proposed, assessing s-IgA concentrations can be instrumental in identifying excessive training workloads and determining URTI risk among professional athletes. The investigation by Tiernan et al. [68] aimed to explore the relationship between s-IgA levels and training load, hypothesizing an inverse relationship. Although no significant associations were found (*p* < 0.005), the study observed a marked increase in training load preceding the decrease in s-IgA levels, suggesting that appropriate training load management and sufficient recovery might mitigate the decline in s-IgA [83]. Lindsay et al. [54] also found a correlation between s-IgA, neopterin, and total neopterin secretion rates and player load. These findings, along with other studies included in this review, indicate that reductions in s-IgA are associated with increased training intensity/volume and congested schedules [48,49,66]. Longitudinal monitoring of training/match loads and mucosal immune function during initial recovery phases can significantly enhance athlete preparation and well-being management strategies. Chronic suppression of salivary mucosal immunity, therefore, can serve as an indicator for necessary workload adjustments to foster athlete well-being.

### 4.4. Inflammatory Markers and Oxidative Stress Markers

The accumulated scientific evidence indicates that periods of fixture congestion coupled with limited recovery results in cumulative match fatigue and amplified physiological strain. This is reflected in unresolved perturbations in inflammatory and oxidative stress biomarkers across successive competitions [44,47,57,61,62,63,66,68]. For example, one study on professional soccer players reported the highest increases in inflammatory cytokines like TNFα and IL-6 along with muscle damage markers like CK and LDH compared to other sports over a regular season [64]. Similarly, consecutive soccer matches over a 1-week period resulted in continually elevated levels of CRP, CK, cortisol, and oxidative stress markers, which showed more pronounced increases after the second match, indicating increased physiological stress and fatigue due to limited recovery between matches [57].

This trend of unsustained inflammation resulting from insufficient recovery periods between matches is corroborated by other soccer studies as well [55,61]. Elite basketball over a 6-month season [65] and professional handball across a 12-week period [47] also exhibited increases in oxidative stress (e.g., ↑GSSG, ↓ GSH/GSSG ratio by 18–35%) during intensive phases along with mild perturbations in inflammation. Greater perturbations were noted in muscle damage (CK) and oxidative stress (TBARS) in sports with higher eccentric loads like handball and basketball vs. volleyball [64]. These highlights varied biochemical demands between sports. Nonetheless, continuous travel and competition without complete inflammatory and redox resolution can heighten injury risk [44,55,61].

Specifically, unabated oxidation can impair muscle contractility and damage cell membranes [66]. Moreover, lingering inflammation can exacerbate muscle damage and slow regeneration between matches [79]. As an example, elevated CRP levels post-match significantly correlated with increases in creatine kinase levels 24 h later in elite soccer players [61]. This illustrates the mechanistic interplay between inflammation and secondary muscle damage. Accordingly, continual biochemical monitoring is vital for balancing stress and recovery, especially for sports involving recurrent high-intensity efforts like soccer, basketball, and handball across congested fixture schedules [47,57]. Regular blood draws can enable training load adjustments to calibrate external and internal loads [2]. This helps stimulate targeted physiological adaptations while mitigating the risk of illness, overtraining, and injury during intensive in-season phases—especially under fixture congestion [35,46].

### 4.5. Sex Differences in Chronic Fatigue Monitoring

While this review focused on male professional team athletes, it is important to acknowledge that sex differences play a significant role in chronic fatigue development, manifestation, and monitoring. These differences stem from physiological, hormonal, and metabolic variations between males and females, which can affect biomarker responses and interpretation [2,24].

One of the primary considerations in female athletes is the influence of the menstrual cycle on fatigue and recovery processes. Hormonal fluctuations throughout the menstrual cycle can impact exercise performance, substrate utilization, and recovery capacity [84]. For instance, estrogen has been shown to have a protective effect against exercise-induced muscle damage, potentially leading to different creatine kinase responses in females compared to males [85]. Testosterone, a key biomarker in our review, exhibits significantly different baseline levels and exercise-induced changes between sexes. While both males and females show acute increases in testosterone following intense exercise, the magnitude of change is typically larger in males [63]. This difference necessitates sex-specific reference ranges and potentially different interpretations of the testosterone/cortisol ratio as a marker of anabolic/catabolic balance [55,63].

Inflammatory responses to exercise also show sexual dimorphism. Some studies have reported that females exhibit a different pattern of inflammatory response following exercise, with potentially different patterns of cytokine release compared to males [86]. This could affect the interpretation of inflammatory markers such as IL-6 and TNF-α in the context of chronic fatigue monitoring. Oxidative stress responses to exercise may also differ between sexes, with some research suggesting that females may have different antioxidant responses compared to males [66]. This could influence the interpretation of oxidative stress markers in fatigue-monitoring protocols. Additionally, differences in muscle fiber composition and metabolism between males and females [87] may affect the accumulation of fatigue and the time course of recovery, potentially necessitating different monitoring strategies and interpretations of biomarker data.

These sex-based differences highlight the need for careful consideration when applying fatigue monitoring protocols developed primarily in male populations to female athletes. Establishing sex-specific reference ranges for key fatigue biomarkers and investigating whether different monitoring strategies are needed for male and female athletes in team sports is necessary [2,24].

### 4.6. Limitations and Future Research Directions

This systematic review has some limitations that should be acknowledged. First, the included studies varied considerably in their methodologies, sample sizes, and specific biomarkers examined, which limited direct comparisons in some cases. Additionally, most studies focused on male athletes, with limited data on female athletes. The review was also restricted to team sports, potentially limiting generalizability to individual sports. Future research should address these gaps by conducting more studies on female athletes and expanding to a wider range of sports. Longitudinal studies tracking biomarker responses across multiple seasons would provide valuable insights into long-term adaptations. There is also a need for more research examining the interactions between multiple biomarkers simultaneously, as well as investigating newer, potentially more sensitive biomarkers. Future studies should aim to establish sport-specific and position-specific reference ranges for key biomarkers to enhance interpretation. Finally, research integrating biomarker data with other monitoring tools like GPS metrics, subjective wellness measures, and performance indicators would provide a more comprehensive understanding of athlete fatigue and recovery processes. Such holistic approaches could lead to more individualized and effective load management strategies in elite team sports.

## 5. Conclusions

This systematic review provides a comprehensive synthesis of the scientific literature on the biochemical monitoring of fatigue in male professional team athletes. The evidence conclusively demonstrates that the high physiological loads imposed by intensive training and match congestion elicit significant alterations in all assessed biomarkers. These indicate measurable muscle damage, oxidative stress, inflammation, immunosuppression, and hormonal strain. Moreover, changes are consistently larger after official matches relative to regular training across sports. Reported recovery kinetics range widely from 24 h to several days post-exercise depending on context.

Overall, this review highlights the utility of frequent biochemical monitoring to quantify biochemical aspects of fatigue alongside sports performance assessments in high-level athletes. This enables coaches to calibrate training stimulus and recovery to stimulate optimal adaptation at the individual level while mitigating injury, illness, and overtraining risks. A key insight is the crucial need for holistic monitoring strategies encompassing both physiological and perceptual indicators of fatigue and the adaptive state. This allows for adjusting external and internal loads to augment performance across a season. Further research should address the impact of psychological stressors alongside physical load metrics for a more complete perspective, particularly on the inflation of baseline biomarker levels as an additional technical error. Nonetheless, this review re-emphasizes biomarker assessment as an invaluable tool for training load management and performance optimization in high-performance sports programs.

## Figures and Tables

**Table 1:** Methodological risk of bias assessment using MINORS checklist.

Study	1	2	3	4	5	6	7	8	9	10	11	12	Score
Barcelos et al. (2017) [44]	2	2	1	1	0	2	2	0	2	2	1	2	17/24
Birdsey et al. (2019) [45]	2	2	1	2	2	2	2	-	-	-	-	1	14/16
Botonis et al. (2023) [46]	1	2	1	1	2	1	2	0	2	1	0	1	14/24
Bresciani et al. (2010) [47]	2	2	2	2	1	1	2	0	2	2	2	2	20/24
Coad et al. (2015) [48]	1	2	1	1	2	1	2	0	2	1	0	1	14/24
Coad et al. (2016) [49]	2	2	2	2	1	2	2	-	-	-	-	2	15/16
Cormack et al. (2008) [50]	2	2	1	2	2	2	2	-	-	-	-	1	14/16
Cunniffe et al. (2011) [51]	2	1	2	2	1	2	2	-	-	-	-	2	14/16
Horta et al. (2019) [52]	2	2	2	2	1	1	2	0	2	2	1	1	18/24
Kamarauskas et al. (2023) [53]	2	2	2	2	1	1	2	0	2	2	2	2	20/24
Lindsay et al. (2015) [54]	2	2	2	2	1	1	2	0	2	2	1	1	18/24
Marin et al. (2013) [55]	2	2	2	2	1	2	2	0	2	1	2	2	20/24
Martínez et al. (2010) [56]	2	2	2	2	1	1	2	0	2	2	1	1	18/24
McLean et al. (2010) [30]	2	2	1	1	0	2	2	0	2	2	1	2	17/24
Miloski et al. (2016) [20]	2	2	2	1	0	2	2	-	-	-	-	2	13/16
Mohr et al. (2016) [57]	1	2	2	1	2	1	2	-	-	-	-	1	12/16
Moreira et al. (2009) [58]	2	2	2	2	0	2	2	0	2	0	1	1	16/24
Moreira et al. (2013) [59]	1	1	2	2	1	2	2	0	2	0	2	1	16/24
Rowell et al. (2018) [60]	2	2	2	2	1	0	2	-	-	-	-	1	12/16
Saidi et al. (2022) [61]	2	2	2	2	1	2	2	-	-	-	-	2	15/16
Schelling et al. (2009) [62]	2	2	1	2	2	2	2	-	-	-	-	1	14/16
Schelling et al. (2015) [63]	2	2	2	2	1	2	2	-	-	-	-	2	15/16
Souglis et al. (2015) [64]	2	2	2	2	1	2	2	-	-	-	-	1	14/16
Spanidis et al. (2016) [65]	2	2	1	2	2	2	2	-	-	-	-	1	14/16
Springham et al. (2021) [66]	2	2	2	2	1	1	2	-	-	-	-	1	13/16
Talaee et al. (2017) [67]	2	2	2	2	1	1	2	0	2	2	2	2	20/24
Tiernan et al. (2020) [68]	2	2	1	2	2	2	2	-	-	-	-	1	14/16
Twist et al. (2012) [69]	2	2	2	2	0	2	2	-	-	-	-	2	14/16

**Note.** The MINORS checklist asks the following information (2 = High quality; 1 = Medium quality; 0 = Low quality): 1. Clearly defined objective; 2. Inclusion of patients consecutively; 3. Information collected retrospectively; 4. Assessments adjusted to objective; 5. Evaluations carried out in a neutral way; 6. Follow-up phase consistent with the objective; 7. Dropout rate during follow-up less than 5%; 8. A control group having the gold standard intervention; 9. Contemporary groups; 10. Baseline equivalence of groups; 11. Prospective calculation of the sample size; and 12. Appropriate statistical analysis.

**Table 2:** Characteristics of the selected studies.

Study	Sample	Period	Type	Test	Frequency of Tests	Results	Conclusions
Barcelos et al. (2017) [44]	8 elite male futsal players (Age: 25.5 ± 5.4 years)	Preseason and season	Matches and training	Muscle damage markers CK and LDH. Oxidative stress markers (IMA and AOPP)	At 3 points in time: End of preseason (T1) Two weeks before Intercontinental Cup (T2) End of season (T3)	Lower values of CK (271–413 vs. 446–777 U/L) and LDH (175–232 vs. 359 441 U/L). Seasonal values of IMA and AOPP.	Biochemical markers can be useful as a means of training monitoring. A one-week fine-tuning period before the main championship (T2) seems to be successful in achieving an optimal state of recovery.
Birdsey et al. (2019) [45]	11 international female netball players (Age: 25 ± 4 years; Mass: 71.8 ± 7.8 kg; Height: 1.8 ± 0.1 m)	During a three-day tournament and the following three days.	Matches	Testosterone, Cortisol, CK	CK measured in the morning of each game day and 62 h post-tournament. Cortisol and testosterone measured at similar intervals.	**Cortisol**: Observed a small and possibly significant decrease on the second tournament day (0.47 µg/dL ± 0.23). On the third day, a trivial, likely non-significant change (0.65 µg/dL ± 0.29). Three days post-tournament, changes were unclear (0.58 µg/dL ± 0.34). **Testosterone**: Registered a small and possibly significant decrease both on the second (102.9 pg/mL ± 25.9) and third days (105.4 pg/mL ± 25.3) of the tournament. Three days later, the decrease remained small and possibly significant (95.7 pg/mL ± 27.0). **Creatine Kinase (CK)**: A very large and likely significant increase on the second (217.2 U/L ± 67.4) and third days (283.0 U/L ± 121.3) of the tournament. Three days later, changes in CK levels were unclear (141.9 U/L ± 113.0)	CK buildup suggests muscle damage during the tournament, with recovery after three days. The decrease in testosterone suggests an influence on performance and motivation. Cortisol showed an initial decrease followed by normalization, indicating adaptation to tournament stress.
Botonis et al. (2023) [46]	8 international water polo players (Age: 28.6 ± 3.9 years; Body mass: 98.9 ± 11.0 kg; Stature: 190.4 ± 6.1 cm)	16 days divided into three phases: PRE-CAMP (3 days before training camp), CAMP (5-day training camp), and POST-CAMP (8 days of congested training and competition).	Training	salivary cortisol, immunoglobulin A	Collection of salivary cortisol, immunoglobulin-A, and subjective wellness measured during PRE-CAMP, CAMP, and POST-CAMP.	In CAMP compared with PRE-CAMP sleep interruptions and salivary cortisol were higher (*p* < 0.01, d = 1.6, d = 1.9, respectively). In POST-CAMP, reduced workload was followed by increased sleep efficiency, reduced sleep disruptions, and moderately affected salivary cortisol; however, overall well-being remained unchanged.	Significant workload increases during a training camp induce sleep disturbances and salivary cortisol increases, which are reversed in POST-CAMP. This suggests that increased workload alongside inadequate recovery affects sleep patterns and may elevate infection risk.
Bresciani et al. (2010) [47]	14 handball male (Age: 20.1 ± 2.5 years)	Preseason and season	Training	Oxidative stress markers (C-reactive protein, GSSG, GSH, and GSH/GSSG ratio)	At 5 time points: Before preseason End of preseason After 1st competition phase (CP) After 2nd CP 7 weeks post-season	Periods of high load: - ↑ GSSG (21.6–38.6 mmol/L). - ↓ GSH/GSSG ratio (18.8–28.9). Positive correlation of GSSG (r = 0.65), GSH/GSSG ratio (r = 0.63) with s-RPE.	Results show that during high-intensity training periods, handball players exhibit minor inflammation and oxidative stress. This highlights the value of closely monitoring psychological and biological markers related to inflammation, oxidative stress, and training load during the season.
Coad et al. (2015) [48]	11 elite male Australian Football League athletes. (Age: 21.8 ± 2.4 years; Height: 186.9 ± 7.9 cm; Mass: 87.4 ± 7.5 kg)	throughout 3 matches during the preseason that were separated by 7 days.	Matches	salivary immunoglobulin A concentration	Saliva samples were collected across each match 24 h and 1 h pre-match and 1, 12, 36, and 60 h post-match.	Across match 3, sIgA was significantly (*p* < 0.01) suppressed at 2 post-match measures (12 and 36 h) compared with pre-match measures (24 and 1 h), which coincided with significantly (*p* < 0.01) elevated player load.	The findings indicated that an increase in player load during the match resulted in compromised post-match mucosal immunological function.
Coad et al. (2016) [49]	18 elite male Australian Football League athletes. (Age: 24 ± 4.2 years; Height: 187.0 ± 7.1 cm; Mass: 87.0 ± 7.6 kg)	16 consecutive matches in an Australian Football League premiership season.	Matches	salivary immunoglobulin A concentration	A concentration (s-IgA) measured at 36 h postmatch throughout an Australian Football League.	Significant (*p* < 0.05) effects compared with baseline sIgA.	Matches may delay sIgA recovery beyond 36 h post-match for full recovery and may be at higher risk of illness during the initial 36 h post-match.
Cormack et al. (2008) [50]	15 elite Australian football League players (Age: 24.9 ± 2.4 years; Height: 1.87 ± 0.07 m; Weight: 88.0 ± 7.9 kg).	Before and during the 22-match season.	Matches and training	Cortisol (C) and Testosterone (T)	Initial data collected at rest approximately 36 h before the first match of the season and on 20 occasions throughout the 22-match season	Cortisol was substantially lower (up to −40 ± 14.1%, ES of −2.17 ± 0.56) than Pre in all but one comparison. Testosterone response was varied, whereas T/C increased substantially on 70% of occasions, with increases to 92.7 ± 27.8% (ES 2.03 ± 0.76).	Change in T/C indicates subjects were unlikely to have been in a catabolic state during the season. Increase in Cortisol compared with Pre had a small negative correlation with performance.
Cunniffe et al. (2011) [51]	31 professional rugby union players (Forwards: *n* = 16, Age: 26.8 ± 0.9 years; Weight: 112 ± 2.6 kg; Height: 188.3 ± 1.7 cm. Backs: *n* = 14, Age: 25.9 ± 0.9 years; Weight: 91 ± 2.0 kg; Height: 182.6 ± 2.4 cm).	48-week competitive season.	Training	Upper respiratory illness (URI), salivary immunoglobulin A (s-IgA), salivary lysozyme (s-Lys), and cortisol.	Weekly illness and TL data were collected during the season. Timed resting morning saliva samples were taken (s-IgA *n* = 11; s-cortisol (*n* = 7) across the season (*n* = 48 weeks).	No significant correlation found between absolute s-IgA or s-Lys concentrations and URI incidence. Peaks in URI were preceded by periods of increased training intensity and reduced game activity. Lower s-IgA (*p* < 0.05) and s-Lys concentrations were consistently observed in backs than forwards, whereas URI incidence also differed for player position (3.4 forwards vs. 4.3 backs). Decreases in absolute s-IgA (December) and s-Lys (November and February) concentrations were associated with a corresponding increase in saliva cortisol (*p* < 0.05).	Regular monitoring of s-IgA and s-Lys may be useful in assessing exercise stress and URI risk status in elite team sport athletes. Stress-induced increases in cortisol release are likely to contribute to reductions in mucosal immunity, predisposing rugby players to increased illness risk.
Horta et al. (2019) [52]	12 elite male volleyball players (Age: 26.9 ± 4.6 years; Body mass: 94.9 ± 11.6 kg; Height: 194.6 ± 8 cm).	A 6-week Short Preparatory Period	Preparatory training sessions	Creatine Kinase (CK), Testosterone (T), Cortisol (Cr), and T/Cr ratio.	Assessments at baseline, after 2nd, 4th, and 6th weeks.	Significant increases in training load and CK levels, indicating muscle damage (r = 0.32; *p* = 0.05) Psychological stress increased, as reflected in the Stress Questionnaire for Athletes (RESTQ-Sport) responses. No significant changes in T, Cr, and T/Cr ratio.	A short preparatory period led to increased training load, muscle damage, and psychological stress without a concurrent increase in physical performance.
Kamarauskas et al. (2023) [53]	21 professional male basketball players (age: 26.2 ± 4.9 years; height: 198.7 ± 6.7 cm; body mass: 93.2 ± 10.0 kg)	5 weeks Pre-season phase	Training and Matches	Testosterone (T), Cortisol (C), and their ratio (T/C)	Saliva samples were collected during an experimental day at the beginning of each week of the preseason phase	No significant (*p* > 0.05) relationships were evident between weekly changes in T, C, or T/C	These results suggest that internal load measures cannot be used to anticipate weekly hormonal responses during the pre-season phase in professional male basketball players.
Lindsay et al. (2015) [54]	24 professional rugby players (Age: 24.2 ± 2.9 years; Mass: 103.3 ± 11.6 kg; Height: 1.87 ± 0.06 m)	3 professional rugby games.	Matches	Myoglobin, salivary immunoglobulin A, cortisol, neopterin and total neopterin	Saliva samples were collected ~120 min pre-game and ~30–40 min post-game.	Post-game decrements (*p* < 0.001), sIgA decreases for game 2 (*p* = 0.019). Mean sIgA decreases following all games.	Significant decreases in sIgA concentration and secretion were observed for game 2. Post-game secretion rate is affected by pre-game rate and number of impacts.
Marin et al. (2013) [55]	10 professional handball players (Age: 25 ± 4.5 years; Mass: 95.3 ± 9.8 kg; Height: 187 ± 6.6 cm)	Over 6 months of competitive season, with evaluations every six weeks.	Matches and training	Oxidative stress and antioxidant biomarkers, muscle damage, biochemical parameters, antioxidant enzymatic activities, functional parameters of immune cells, production of superoxide anion, nitric oxide, and hydrogen peroxide.	Blood samples were collected four times every six weeks throughout the season. At each blood collection (T1–T4), samples were collected in the morning (10 a.m.)	Plasma TBARS: Increased significantly (4.4-fold post-T3, 3.2-fold post-4). Plasma Thiols: Marked decrease during intense periods. Erythrocyte TBARS: Transient rise, significant reduction by T4. Erythrocyte Antioxidant Enzymes: Dramatic increase (up to 14.7-fold for superoxide dismutase at T4). Creatine Kinase: 94% increase after T4, indicating muscle damage. Lactate Dehydrogenase: Decreased, then normalized. IL-1β: Significant decrease post-T2. IL-6 and TNF-α: Stable levels. Lymphocyte Proliferation & Neutrophil Phagocytic Capacity: Notable fluctuations, 20% decrease in phagocytic capacity.	Oxidative stress and antioxidant biomarkers can change throughout the season in competitive athletes, reflecting the physical stress and muscle damage that occurs as the result of competitive handball training. In addition, these biochemical measurements can be applied in the physiological follow-up of athletes.
Martínez et al. (2010) [56]	12 professional basketball players (Age: 25.3 ± 4.4 years; Height: 1.98 ± 0.10 m; Weight: 96.8 ± 13 kg)	Preseason and season	Matches and training	Cortisol and Testosterone	At 4 time points: Preseason (T1) End of 2nd mesocycle (T2) King’s Cup (T3) End of regular season and Eurocup (T4)	Catabolic/anabolic balance throughout the season: Decrease in cortisol levels at T2 and T4. Increase in T/C ratio at T2 and decrease at T3.	Increase in testosterone and decrease or maintenance of cortisol levels can contribute to effective recovery. Monitoring cortisol, testosterone, and training levels is useful to prevent stress and manage recovery periods during the season.
McLean et al. (2010) [30]	12 professional rugby league players (Age: 24.3 ± 3.6 years; Body mass: 101.9 ± 8.4 kg; Stature: 184.7 ± 6.1 cm)	During three different duration training weeks throughout a 26-week rugby league season.	Matches and training	Testosterone, Cortisol, and Testosterone/Cortisol ratio (T/C)	Saliva samples collected 4 h pre-match and 1, 2, and 4 days post-match in all three experimental weeks.	A significantly higher mean daily load was found in the 7-day (*p* < 0.05, d = 0.45) and 9-day (*p* < 0.01, d = 0.59) microcycles compared with the 5-day microcycle. Day 4 cortisol measures in the 9-day and 7-day microcycles were significantly higher than game day (*p* < 0.01, d = 0.60) and tended to be higher than day 1 measures, approaching significance (*p* = 0.07, d = 0.69).	The study highlights the complexity of using salivary hormones, especially testosterone and the T/C ratio, as reliable indicators of fatigue or anabolic/catabolic state in professional rugby players. Cortisol showed some correlation with training load and recovery, but its variability also suggests limitations in its use as a sole indicator of physiological stress or fatigue in this sporting context.
Miloski et al. (2016) [20]	Twelve male professional futsal players (24.3 ± 4.7 years old; 75.5 ± 7.7 kg; and 173.4 ± 4.5 cm)	Preseason and partially (midway) season	Training	Testosterone, Cortisol, and Testosterone/Cortisol ratio (T/C). CK	Every 2 weeks (preseason) and every 4 weeks (season)	Increase in CK (266 μ/L) and T/C ratio (2.0) at the end of preseason. Increase in cortisol (+3.9 mg/dL) and decrease in T/C ratio (−0.5) from Blood samples 4 to Blood Samples 5	During the in-season, players kept their CK values stable without any loss in physical performance, suggesting that stable blood CK levels are a physiological feature of active futsal players. Seasonal hormonal data show futsal players effectively handled training and competition stress, as indicated by the stable T/C ratio without linked performance decline.
Mohr et al. (2016) [57]	40 competitive male soccer players (Age: 21.5 ± 0.3 years; Height: 1.77 ± 0.01 m; Weight: 73.4 ± 0.9 kg)	Congested 1-week study over 11 days, including baseline testing, three 90 min games, and 9 days of practice sessions and testing between and after games.	Matches and training	PC, NEFA, Urea, Ammonia, Glycerol, Adhesion molecule concentrations, CK GSH, GSSG, TBARS, CAT, Cortisol, Testosterone, Cytokines, CRP, TAC.	Venous blood samples collected every morning until the 3rd day after the final match. Additional samples before each match and 3–4 min after the end of the first and second half of each match for metabolite measurement.	Elevated levels of CK, CRP, and cortisol were noted 48 h post-games, with more significant increases following the second match. Oxidative stress markers such as TBARS and carbonylated proteins showed substantial increases post-games. The reduced/oxidized glutathione ratio declined during the first 24 h post-games	Inflammatory and oxidative stress responses to consecutive match microcycles indicate increased physiological stress and more pronounced fatigue after the second game, particularly due to the short three-day recovery period.
Moreira et al. (2009) [58]	22 male professional soccer players (Age: 23 ± 4 years; Height: 182 ± 6.8 cm; Body mass: 78.6 ± 8.4 kg).	During the competitive season.	Competitive training soccer match	Salivary cortisol concentrations	Subjects provided resting saliva samples approximately 10 min before the pre-session warm-up (PRE) and post-session saliva samples were col- lected within 10 min after the conclusion of the match (POST).	No significant changes in salivary cortisol concentrations (*p* > 0.05) were observed between teams or time points. Individual responses varied, showing both increases and decreases in cortisol levels.	The study indicates that a competitive soccer match does not significantly impact salivary cortisol levels in top-level professional soccer players adapted to this type of stress. Highlights the need for individual analysis due to response variability among players.
Moreira et al. (2013) [59]	12 elite Brazilian futsal players (age: 19 ± 1 years; height: 180 ± 4 cm; and body mass: 73 ± 7 kg).	4 weeks of intensive training during the competitive season with 27 training sessions performed.	Training	salivary immunoglobulin A, cortisol, and upper respiratory tract infection (URTI)	Salivary immunoglobulin A, salivary cortisol, and symptoms of URTIs were assessed weekly.	No significant differences were observed for sIgA during the study (*p* > 0.05). The relative change in sIgA absolute was associated with the URTI severity during week 4 (r = −0.74; *p* < 0.05).	Futsal athletes were more susceptible to high URTI symptom severity in periods of higher training intensity and volume. A reduction in training load before competitions is an appropriate strategy to minimize URTI symptoms, ensuring the athlete’s ability to train and compete.
Rowell et al. (2018) [60]	23 elite soccer players (Mean age: 23.3 ± 4.1 years; Height: 180 ± 10.0 cm; Weight: 75.7 ± 4.4 kg)	Competitive season with 34 matches (27 regular league and 7 Asian Champions League matches).	Matches	testosterone, and cortisol	Saliva collection before the last training session prior to match play, between 09:00 and 09:30 a.m., following strict pre-test procedures.	Position-specific responses to training loads. Center defenders showed a reduction in performance ratings with increased load, while strikers and wide midfielders tended to improve with increased load. Wide midfielders also showed increased testosterone levels with increased training load.	Increases in training load significantly affect hormonal levels, especially an increase in cortisol and testosterone in center defenders and changes in the testosterone/cortisol ratio.
Saidi et al. (2022) [61]	14 elite soccer players (Age: 20.9 ± 0.8 years; Height: 177 ± 5 cm; Weight: 72.4 ± 5.2 kg)	12 weeks (T1–T2: 6 regular weeks; T2–T3: 6 congested weeks)	Matches and training	CRP, CK, Creatinine	Evaluations at T1 (week 1), T2 (week 6), and T3 (week 12)	Significant increase in stress, fatigue, DOMS, and Hopper Index during congested period. Notable correlations between Δ% of CRP, Δ% of CK, Hopper Index, and Δ% of fatigue	Increases in biochemical markers and changes in well-being during congested periods indicate a direct relationship with training load. Monitoring these parameters is vital to prevent overtraining and optimize performance
Schelling et al. (2009) [62]	Male professional basketball team (27.8 ± 4.8 years; 97 ± 9.5 kg; 197.2 ± 7.3 cm)	During the competitive season	Matches and training	Testosterone, Cortisol, and Testosterone/Cortisol ratio (T/C).	4–6 weeks (8 Samples)	Increase in cortisol in preseason (+33%) and maintained throughout the season (0.393–0.516 mmol/L). Increase of testosterone after 3.5 days of rest and decrease at the end of the season (20.6–24.9 vs. 18.0 mmol/L). T/C ratio decreases at the end of the season (48.0–61.7 vs. 35.4).	The T/C ratio and/or testosterone could be used as an indicator of the state of be used as indicators of recovery status and help to optimize individualized individualized training loads to avoid episodes of excessive fatigue.
Schelling et al. (2015) [63]	20 professional male basketball players	Four consecutive seasons	Regular season matches and training	Blood plasma total testosterone (TT) and cortisol (C) levels, testosterone-to-cortisol ratio (TT/C).	Blood samples were collected periodically every 4–6 weeks. Always after a 24- to 36-h break after the last game played.	Hormonal levels in professional basketball players were found to be position-dependent. Power forwards (PF) showed the lowest total testosterone (TT) levels (median 18.1 ± 4.9 nmol/L), while small forwards had the highest cortisol levels (0.55 ± 0.118 mmol/L). Players with 13–25 min of game time per match exhibited the highest TT (22.8 ± 6.9 nmol/L) and TT/C ratios (47.1 ± 21.2). The most stressed hormonal state, characterized by low TT/C and high cortisol levels, was observed in March and April, highlighting the need for tailored management based on playing time and position.	Monitoring plasma TT and cortisol is crucial for managing stress induced by the demands of a professional basketball season. Hormonal status varies according to playing position and game time, impacting training and recovery strategies.
Souglis et al. (2015) [64]	72 elite male players from four team sports (soccer, basketball, volleyball, handball).	Start of the regular season.	Matches.	TNFα, IL-6, CRP, CK, LDH, Urea, Ammonia, Cortisol	Pre, post, 13 h, and 37 h after the match.	Soccer showed the highest increase in inflammatory cytokines and muscle damage markers. Volleyball showed the least increase compared to the other three sports.	Professional soccer matches impose higher metabolic demands and cause greater inflammatory responses and muscle damage compared to handball, basketball, and volleyball.
Spanidis et al. (2016) [65]	14 adult male basketball players (age, 26.8 ± 1.2 years; height, 1.99 ± 0.02 m; weight, 101.6 ± 2.63 kg)	Internal Season	Regular season matches and training.	Markers of oxidative stress (TAC, TBARS, GSH, CARB, and sORP)	Beginning and end of season	Increase in sORP values (200 vs. 220 mV; 9.6%) and TAC (0.8 vs. 1.0 mmol/L; 12.9%) at the end of the season. Decrease in GSH (3.7 vs. 2.5 mmol/g; 35%) at the end of the season. Large inter-individual variation in TBARS, CARB of TBARS, CARB, TAC. High correlation between sORP and CARB (r = 0.798).	The sORP can help to monitor the redox status of a group of the redox status of a group of athletes, with higher greater completeness than TAC. An individualized examination of the redox status through TBARS, CARB, and TAC is required to identify critical recovery periods for each athlete.
Springham et al. (2021) [66]	18 senior professional male soccer players (Age: 24 ± 3.8 years; Height: 181 ± 7.0 cm; Body mass: 72.4 ± 5.2 kg).	A 6-week preseason and a 40-week season, divided into eight 5-week mesocycles.	Regular season matches and training.	Salivary immunoglobulin-A (s-IgA), α-amylase (s-AA), testosterone (s-T), cortisol (s-C), and testosterone/cortisol ratio (s-T/C); Athlete Self-Report Measures (ASRM) including fatigue, sleep quality, and muscle soreness.	Bi-weekly collection following recovery days	The study found small reductions in salivary immunoglobulin-A (*p* = 0.003), *α*-amylase (*p* = 0.047), and cortisol (*p* = 0.007), with trivial changes in testosterone. The testosterone/cortisol ratio varied inversely with workload. Self-reported fatigue, sleep quality, and muscle soreness improved across the season’s first half (*p* = 0.030 to *p* = 0.005, small effect). Hormonal changes correlated with self-reports (R^2^ = 0.43 to 0.45), with cortisol linked to worse and testosterone/cortisol to better reports. Non-linear relationships were found between some hormones and self-reports, indicating optimal levels for best responses.	Chronic suppression of mucosal immunity was observed. Salivary measures related to self-report measures, indicating the need for reduced workload to improve wellbeing. Monitoring s-IgA, s-T, s-C, and s-T/C can be effective in assessing players’ health and performance status.
Talaee et al. (2017) [67]	15 elite male basketball players (Age: 24 ± 1.5 years; weight: 83 ± 3.3 kg; height: 188 ± 6.1 cm)	6 weeks	Combined training	RBC, Hb, Hct, MCV, MCH, WBC, PLT, MCHC	Pre-, post-, and 24 h after training	Significant increase in white blood cell and platelet counts at two stages; post-training and 24 h after recovery. Hemoglobin, hematocrit, and red blood cell count significantly decreased after 24 h of recovery.	Combined training plays a significant role in physiological and hematological adaptation processes, enhancing athletic performance. Prescribing this training method should be coupled with regular blood biochemical monitoring to balance exercise stress and recovery strategies.
Tiernan et al. (2020) [68]	19 male elite rugby union players.	over a 10-week training period	Training	Upper respiratory illness (URI), salivary immunoglobulin A (s-IgA)	Saliva samples were collected twice a week, Monday and Friday, within 1 h of the players waking up before training.	No significant differences in weekly sIgA levels were found over the 10-week period. The likelihood of suffering from a URTI increased when sIgA significantly decreased (*p* = 0.046).	A decrease in >65% of sIgA meant players were at risk within the following 2 weeks of contracting a URTI.
Twist et al. (2012) [69]	23 male rugby league players. Players were categorized as backs (*n* = 10; age 25.9 ± 5.1 years; stature 1.82 ± 0.08 m; body mass 91.9 ± 11.6 kg) or forwards (*n* = 13; age 26.0 ± 4.1 years; stature 1.83 ± 0.06 m; body mass 102.0 ± 6.7 kg)	European Rugby League Super League season	Matches	Creatine Kinase (CK)	Assessments conducted pre-match, one day after (day 1), and two days after the match (day 2).	Creatine kinase was higher both 1 and 2 days after than before matches (*p* < 0.05) in forwards and backs.	Despite the mechanisms of fatigue being different between forwards and backs, our results highlight the multidimensional nature of fatigue after a rugby league match and that markers do not differ between positions.

**Note:** AOPP (Advanced Oxidation Protein Products), ASRM (Athlete Self-Report Measures), CARB (Protein Carbonyls), CAT (Catalase Activity), CK (Creatine Kinase), CRP (Plasma C-Reactive Protein), Cortisol (C), Doms (Delayed Onset of Muscle Soreness), GSH (Reduced Glutathione), GSSG (Oxidized Glutathione), HB (Hemoglobin), Hct (Hematocrit), IL-6 (Interleukin-6), IMA (Ischemia Modified Albumin), LDH (Lactate Dehydrogenase), MCH (Cell Hemoglobin), MCHC (Mean Red Cell Hemoglobin Concentration), MCV (Mean Red Cell Volume), NEFA (Non-Esterified Fatty Acids), PC (Protein Carbonyls), PLT (Blood Platelets), PF (Power Forwards), RBC (Red Blood Cells), s-IgA (Salivary Immunoglobulin-A), TAC (Total Antioxidant Capacity), TBARS (Thiobarbituric Acid-Reactive Substances), TL (Training Load), TNFα (Tumor Necrosis Factor-a), T (Testosterone), T/C (Testosterone/Cortisol Ratio), TT (Blood Plasma Total Testosterone), URI (Upper Respiratory Illness), WBC (White Blood Cell), hs-CRP (High-Sensitivity C-Reactive Protein), s-AA (α-Amylase), s-C (Salivary Cortisol Concentrations), s-Lys (Salivary Lysozyme), and sORP (Static Oxidation-Reduction Potential Marker).

**Table 3:** Summary of key biomarkers for chronic fatigue assessment in professional team sport athletes.

Biomarker Category	Specific Biomarkers	Relevance to Chronic Fatigue	Typical Measurement Method
*Muscle anabolic/catabolic hormones*	Testosterone, Cortisol, Testosterone/Cortisol ratio	Reflect metabolic strain and stress responses to training/competition loads	Blood or saliva samples; ELISA or radioimmunoassay
*Muscle damage markers*	Creatine Kinase (CK), Lactate Dehydrogenase (LDH)	Quantify extent of exercise-induced muscle damage	Blood samples; Spectrophotometry
*Immunological markers*	Salivary Immunoglobulin A (s-IgA), Immune cell function	Indicate mucosal immunity status and potential vulnerability to upper respiratory tract infections	Saliva samples; ELISA
*Oxidative stress markers*	Reactive Oxygen Species, Antioxidant Capacity, TBARS, Protein Carbonyls	Assess cellular stress and redox balance	Blood samples; Spectrophotometry, ELISA
*Inflammatory markers*	C-Reactive Protein (CRP), Cytokines (e.g., IL-6, TNF-α)	Indicate systemic inflammatory responses to prolonged intense training	Blood samples; ELISA, Flow cytometry

**Note.** ELISA: Enzyme-Linked Immunosorbent Assay; TBARS: Thiobarbituric Acid Reactive Substances; IL-6: Interleukin-6; TNF-α: Tumor Necrosis Factor-alpha.

## Data Availability

Not applicable.
